# Malaria prevention and care seeking among gold miners in Guyana

**DOI:** 10.1371/journal.pone.0244454

**Published:** 2020-12-29

**Authors:** Bolanle Olapeju, Camille Adams, Gabrielle Hunter, Sean Wilson, Joann Simpson, Lyndsey Mitchum, TrishAnn Davis, Jennifer Orkis, Horace Cox, Neil Trotman, Helen Imhoff, Douglas Storey

**Affiliations:** 1 Johns Hopkins Center for Communication Programs, Baltimore, Maryland, United States of America; 2 Breakthrough ACTION Guyana, Georgetown, Demerara-Mahaica, Guyana; 3 National Malaria Program, Ministry of Health, Georgetown, Demerara-Mahaica, Guyana; Ministry of Health and Sports, Myanmar, MYANMAR

## Abstract

Despite being a priority population in malaria elimination, there is scant literature on malaria-related behavior among gold miners. This study explores the prevalence and factors influencing malaria prevention, care seeking and treatment behaviors in Guyana gold mining camps. A cross sectional survey was conducted among adult gold miners living in mining camps in the hinterland Regions 1 (Barima-Waini), 7 (Cuyuni-Mazaruni), and 8 (Potaro-Siparuni). Multivariable logistic regressions explored factors associated with miners’ self-report of mosquito net use, prompt care-seeking; self-medication; and testing for malaria. A third of miners used a mosquito net the night preceding the survey and net use was higher among those who believed that net use was the norm in their camp (aOR: 3.11; 95% CI:1.65, 5.88). Less than half (45%) of miners had a fever in the past 12 months, among whom 36% sought care promptly, 48% tested positive for malaria while 54% self-medicated before seeking care. Prompt care-seeking was higher among miners with high malaria knowledge (aOR: 1.44; 95% CI: 1.01, 2.05). Similarly, testing rates increased with secondary education (aOR: 1.71; 95% CI: (1.16, 2.51), high malaria knowledge (aOR: 1.45; 95% CI: 1.02, 2.05), positive beliefs regarding malaria transmission, threat, self-diagnosis, testing and treatment, and, trust in government services (aOR: 1.59; 95% CI (1.12, 2.27) and experience of a prior malaria episode (aOR: 2.62; 95% CI: 1.71, 4.00). Self-medication was lower among male miners (aOR: 0. 52; 95% CI: 0.32, 0.86). Malaria prevention and care seeking behaviors among miners are somewhat low and influenced by mosquito net usage, perceived norms, malaria knowledge and prior episode of confirmed malaria. Study findings have implications for malaria interventions in the hinterland regions of Guyana such as the mass and continuous distribution of insecticide treated nets as well as community case management initiatives using trained malaria testing and treatment volunteers to curb malaria transmission among remote gold mining populations. These include efforts to identify and address gaps in distributing mosquito nets to miners and address miners’ barriers to prompt care seeking, malaria testing and treatment adherence. Targeted social and behavior change messaging is needed on net acquisition, use and care, prompt care-seeking, malaria testing and treatment adherence. Additional efforts to ensure the overall sustainability of the community case management initiative include increased publicity of the community case management initiative among miners, use of incentives to promote retention rates among the community case management volunteer testers and public private partnerships between the Guyana Ministry of Health and relevant mining organizations.

## Introduction

In Guyana, malaria transmission remains concentrated among mining populations in the hinterland Amazonian Regions 1 (Barima-Waini), 7 (Cuyuni-Mazaruni), 8 (Potaro-Siparuni), and 9 (Upper Takutu- Upper Essequibo) [1]. Historically, these regions are classified as Guyana’s malaria endemic regions, since they account for 85–95% of the total malaria cases. According to the 2019 World Malaria Report, 11% of Guyana’s population of about 746,955 people lives in these malaria high transmission areas (more than 1 case per 1000 population). Inhabitants of these regions engage in gold mining and logging as their main economic activities. Largely unpopulated, the hinterland regions are primarily made up of Amazon forest while the majority of the population (89%) lives in the coastland regions [2].

**Fig 1:**
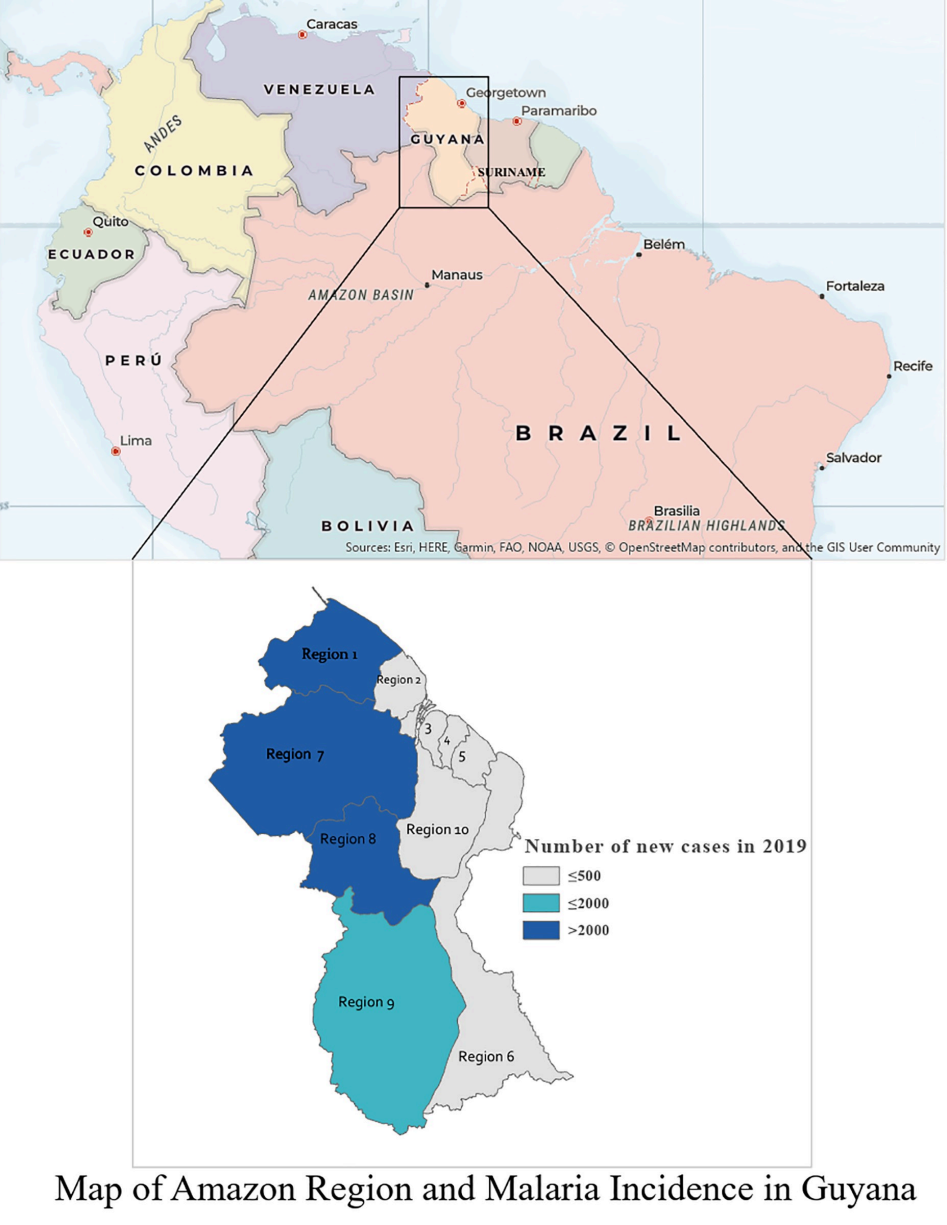


Although there has been an overall reduction in malaria cases from 1996–2019 [3], the period was characterised by peaks in cases in 1998 (50,000+ cases), 2005 (~40,000 cases), 2012 (~ 32,000 cases), and 2018 (16,500+ cases). From 2013 to 2015 malaria cases decreased by 68.3% (21,495 cases), but then rose again from 2015 to 2019. To reduce malaria prevalence, the Ministry of Health (MoH) through Vector Control Services (VCS) renewed efforts to increase access to diagnosis, treatment, and vector control interventions such as long lasting insecticide treated nets (LLINs); emphasize social and behavioural change; conduct Indoor Residual Spraying (IRS) to respond to outbreaks as required; and improve surveillance. Fig 1 highlights the current malaria incidence in Guyana as of 2019.

*Anopheles darlingi* is the principal vector that transmits malaria in Guyana, particularly in the hinterlands, since it is mainly a riverine mosquito generally confined to rural, lowland forested locations [4]. *Anopheles aquasalis* is considered a possible secondary vector, primarily in the coastal areas due to its tolerance to saltwater environments and distribution along the Atlantic Coast [4,5]. The larval habitats of the *Anopheles* mosquito are natural water bodies such as lagoons, lakes, and particularly slow-flowing streams or rivers with clear shaded water. The rich deposits of gold and diamonds within the hinterland region attract a large number of mining operations [6]. Mining in the hinterland is significantly associated with malaria in Guyana as it contributes to deforestation and other environmental changes that create habitats favorable for *Anopheles* mosquitoes [7].

An increase in the price of gold corresponds to a rise in mining operations throughout the country. Many people migrate from the coastal areas in Guyana to the hinterland to engage in mining and related activities. This increases the number of susceptible people at risk of malaria. The increase in the number of at-risk populations subsequently translates to peaks in the number of malaria cases during periods or years when the price of gold peaks. The prevalence of malaria is higher in areas that border with Venezuela and Brazil and also among illegal miners from foreign countries [3].

Miners are a priority group for malaria control as they are a migrant and hard-to-reach population with limited access to health care services [3,8]. Many miners and loggers are treated outside of the public health system, resulting in inadequate malaria data collection captured by the national health management information system [3]. Miners are reservoirs for malaria [9–11] and are at risk of other adverse health outcomes due to factors such as low levels of education, biomass fuel smoke exposure, tobacco smoking, higher alcohol consumption, and unsafe sexual practices [12–14]. Several health-related behaviors are needed for malaria control, particularly among miners [15,16]. These include prompt care-seeking within 24 hours of onset of malaria symptoms, malaria testing and adherence to test results by using malaria treatment only after a positive malaria test, treatment with only approved medications after a positive test, completing malaria treatment and not self-medicating with over-the-counter drugs [9,12]. However, there is limited data regarding these behaviors among miners in Guyana [8,17]. A 2014 survey of fifty patients at a public malaria clinic in Guyana [18] showed that 26% of patients self-diagnosed, and 74% had tried self-medication at home with antimalarial drugs, pain killers, or herbal or “bush” medicines. Furthermore, 48% of the respondents were only partially knowledgeable about malaria, while 14% were unaware of malaria.

The USAID funded Breakthrough ACTION Guyana project is working in collaboration with the Guyana MoH, the Pan American Health Organization (PAHO), and the Global Fund to Fight AIDS, Tuberculosis and Malaria (GFATM) to implement innovative evidence- and theory-based Social and Behavior Change (SBC) interventions to support community case management of the malaria program. Regions 1, 7 and 8 are intervention sites for the Breakthrough ACTION Guyana project. With support from PAHO and the World Health Organization, MoH developed a community case management volunteer testing program to support greater access to malaria services by miners in Regions 1, 7, 8, and 9. The program focuses on recruitment and training of stable workers from mining camps, such as cooks, camp managers, security guards and shop owners in the communities. Volunteers are trained to perform malaria rapid diagnostic tests (RDTs) on symptomatic persons and to provide treatment for individuals with uncomplicated cases of malaria, except for children and pregnant women. Thus, miners with symptoms of malaria present to the volunteer testers who provide counselling, testing and treatment services as needed. After the training, the RDT kits and treatment are provided to the volunteers and regional VCS staff are responsible for monitoring and restocking the supplies. Stock-outs may occur due to delays in the country’s procurement system and challenges with travel in the interior.

The community case management initiative was first piloted in Region 8 in April 2016 and then officially rolled out in Regions 1, 7, 8, and 9 in 2017. Since then, the National Malaria Program (NMP) has conducted numerous volunteer trainings. Breakthrough ACTION supported the development of the training curriculum and piloted branded materials, such as flags and certificates, Regions 7, and 8 to increase the visibility of the community case management program. The Breakthrough ACTION project supported the development and distribution of counseling materials in Regions 1, 7 and 8, to assist testers in providing accurate information.

### Conceptual considerations

There is a great need for research on malaria-related behavior among miners in Guyana [19] to inform the National Malaria Program Strategy [3] and the ongoing malaria community case management program to the use of long-lasting insecticidal mosquito nets, promote prompt care-seeking for malaria, and improved malaria testing uptake [3]. Understanding current individual behaviors around malaria care-seeking, testing, treatment, and prevention will enable the National Malaria Program and its stakeholders to design relevant programs to improve malaria outcomes among miners.

This study was not intended to test particular hypotheses about causes of bed net use or testing and treatment adherence. Its goal was to explore the prevalence and importance of factors influencing malaria care-seeking and treatment behaviors in the gold mining camps of Regions 1, 7, and 8 in Guyana to inform the design of relevant SBC interventions and other efforts to promote the sustainability of the community case management initiative. Specific desirable behaviors within the mandate of the MoH include prompt care-seeking within 24 hours of symptom onset, self-medication versus adherence to the use of government approved treatments, testing for malaria, and the use of long-lasting insecticidal mosquito nets.

Although it does not propose specific hypotheses, this exploratory study is nevertheless guided by a conceptual framework—the Ideational Meta-Theory of Communication (Kincaid et al., 2007)—that has proven to be useful in other malaria-related studies of mosquito net use [20], as well as in other areas of health behavior: adoption of contraception [21], improved HIV prevention [22] and improved Ebola response [23], among others. The ideation framework recognizes that most behavioral decisions are driven by multiple psychosocial variables, often simultaneously. It incorporates multiple cognitive, emotional and social constructs from various behavioral theories, including knowledge of disease symptoms, transmission and prevention, beliefs, values, and attitudes related to proposed actions, emotional variables, such as perceived severity and susceptibility to disease, perceived social norms around proposed actions; perceived self-efficacy and belief in the efficacy of proposed actions; as well as cognitive variables, such as social support, social influence, spousal/partner communication, and personal advocacy. The combination of variables that explain behavior in a particular context are determined by the sociocultural setting and social interaction within a given community, thus it tends to be location specific. Ideational analysis uses a combination of bivariate and multivariable regression to identify the unique combination of variables that informs decision making in a particular place, but that also generalizes best to a population of interest, in this case, gold miners in certain regions of Guyana.

## Materials and methods

### Study design and population

The study design was a cross sectional quantitative survey among adult miners between the ages of 18–59 years living in mining camps in Regions 1, 7, and 8. These regions are malaria prone regions of Guyana and intervention sites for the community case management malaria program as well as the Breakthrough ACTION Guyana project. The study was conducted in November to December 2019 as a baseline survey for the USAID funded Breakthrough ACTION Guyana project. The survey objective was to inform the design of relevant SBC interventions to improve malaria related outcomes among miners in these regions.

### Sample size calculations

An estimated sample size of 1630 miners was calculated based on an estimated proportion of 47% of miners that currently self-diagnose and self-treat, an estimated 5 percentage point decrease (delta) in this proportion by the end of the Breakthrough ACTION Guyana project, a 95% confidence interval, power of 80% and non-response rate of 5%.

### Sampling

Prior to data collection, the study conducted a verification exercise in November 11–18, 2019 to identify the number of miners within the study regions. Earlier data collected from the Guyana Geology and Mining Commission accessed in July 2018 estimated that there were about 16,010 licensed miners in the study regions. However, this list only detailed licensed miners but not the miners that were actively mining, did not account for the transitory nature of mining work and did not include small or unlicensed camps and miners. Furthermore, in 2019, there were major fluctuations in the price of gold and this resulted in fewer miners actively mining for gold. Anecdotally, when the price of gold drops, many miners stop working temporarily as they are less likely to make a profit due to the prohibitive costs of mining. The verification was also conducted because mining activities and miners tend to migrate often to new camps and areas.

The verification exercise was implemented with sensitization activities in addition to visits to some camps and meetings with local community experts to verify the mining camp locations and numbers. Regional coordinators made site visits to central hubs in each survey region to ascertain the types of mining facilities available, to verify the presence of miners in the sampled areas, and to meet with key stakeholders in the regions to inform them of study activities. Study staff also met with Guyana Geological and Mining Commission officers responsible for the sampled areas in each region, who shared their most current numbers of miners in the proposed locations. This updated information, presented in Table 1, was used to compile the final sample listing in Regions 1, 7, and 8. However, the verification process was not exhaustive. Of the 165 mining areas identified for verification, 117 mining areas were verified as operative, 20 mining areas were confirmed as inactive, while about 28 areas were not verified due to security concerns, inaccessibility or prohibitive transportation costs.

**Table 1:** Final distribution of study participants in Regions 1, 7 & 8.

Region	Large camps (>22 miners)		Medium camps (8–22 miners)		Small camps (<8 miners)		Total (% total)	
	# camps	# miners	# camps	# miners	# camps	# miners	# camps	# miners
1	6	113	23	231	13	46	42	**390 (23.2%)**
7	10	142	61	495	47	182	118	**819 (48.6%)**
8	9	144	31	212	33	120	73	**476 (28.3%)**
**Total**	**25**	**399 (23.4%)**	**115**	**938 (55.7%)**	**93**	**348 (20.6%)**	**233 (100.0%)**	**1685 (100.0%)**

A total of 4051 miners were verified across Regions 1 (795 miners), 7 (2486 miners), and 8 (770 miners). A sampling frame was developed based on the mining location and the number of miners in the different camp sites across the regions. Typically, the size of mining camps is determined by the type, number and level of sophistication of machinery as these directly influence production levels. For this study, the size of mining camps was based on the number of miners. Large camps were defined as camps with 23 miners or more, medium camps had 8–22 miners while small camps had seven or less miners. In camps identified as large or medium it was possible that smaller operations were found instead of either one large or one medium sized mining camp. The final sampling frame of full list of mining locations and mining camps is included in S1 Table. S2 Table shows the sampling implementation summary showing number of miners verified, approached and interviewed.

In executing the sample selection in the field, if the first camp visited did not have enough miners to provide the full quota for the area, all the miners at this first camp were approached for an interview. No random sampling of persons to interview was required in this case. In the next camp visited, the outstanding number of miners for the area quota was then interviewed. Random selection was needed if the number of miners at this second camp exceeded the balance needed for the area quota. If the number of miners at the second camps was less than the balance needed, all the miners at this second camp were interviewed before moving to the third camp and so on until the area quota was obtained.

### Ethical considerations

The study received institutional review board approval from the Johns Hopkins Bloomberg School of Public Health and the Guyana Ethical Review Committee. All respondents provided written consent prior to participating in the survey.

### Data collection

The survey questionnaire was pretested and validated on November 19, 2019 after a two day training of the data collectors (November 17–18, 2019). Data collection started on the 20th of November and lasted until the 5th of December. For miners who provided informed consent to participate, surveys were conducted face-to-face in the mining camp in a quiet location where their responses were not overheard by other miners. The interviews lasted about 20 minutes on average. The questionnaire included the following sections: Mining camp identification, Personal Information, Knowledge about malaria, Treatment and Testing Attitudes and Behavior, Use of Insecticide treated mosquito net, and, Exposure to information about malaria testing and treatment. The questionnaire for the survey was formatted for use on tablets in offline mode using the Kobo Toolbox platform. Tablets were used in the pilot and this allowed further testing of the instrument. No major issues were identified with the instrument.

The final number of recruited miners was 1685 and the distribution is shown on Table 1.

### Data analysis

Key behavioral outcomes included miners self-report of the following:

1. Mosquito net use—defined as sleeping under a mosquito net the night preceding the survey;
2. Prompt care-seeking—defined as seeking advice or treatment the same day or the next day after the onset of fever symptoms;
3. Self-medication—defined as taking any medication for the fever before seeking advice or testing;
4. Testing for malaria—defined as having blood taken for testing at any time during the fever.

Prompt care seeking and testing for malaria was not specific to services provided by the community case management program but reports on the miners’ uptake of services regardless of the provider.

Additional behavioral outcomes assessed in the study included miners’ self-report of any care seeking regardless of duration from onset of symptoms (no versus yes), testing positive for malaria (no versus yes), being prescribed treatment after a positive malaria test (no versus yes), receiving prescribed medications (no versus yes) and completing prescribed medications (no versus yes).

Other covariates of interest included sociodemographic characteristics such as age in years (less than 35 versus 35 or more years old), sex (male versus female), region (Region 1, 7 or 8), education (No education, some primary, completed primary, some secondary, beyond secondary), marital status (not married versus married), mining experience (less than five versus five or more years), exposure to malaria prevention messages (no versus yes), number of malaria episodes in the past year (one, two, three or more), high malaria knowledge (measured as a composite score of 11 (the median score) or more based on responses to 20 questions on malaria, its causes, types, symptoms, testing, side effects and prevention measures), miners’ beliefs (measured as a composite score of 15 (the median score) or more based on responses to 20 Likert-scale questions on their attitudes towards malaria transmission, threat, self-diagnosis, testing and treatment, and, trust in government services related to malaria).

Chi-square, t-tests and ANOVA were used for bivariable tests of association and exploratory analysis. Data analysis regarding mosquito net use was limited to miners who had a mosquito net at the time of the survey, while data analysis regarding prompt care-seeking, self-medication, and malaria testing was limited to miners with at least one episode of fever in the 12 months preceding the survey. Multivariable linear and logistic regressions were also employed to explore factors associated with the key outcomes of the study. The covariates included in the regression models were identified from SBC theory (including knowledge, beliefs, perceived norms) as well as apriori knowledge of the mining context in Guyana (miners’ sociodemographic characteristics). Data management and analysis was done using Stata version 16 (Stata Corporation, College Station, TX, USA) and Excel 2016 (Microsoft Corp, Seattle, WA, USA). The data was weighted using the *svyset* command in STATA to account for clustering of miners at the camps and to make the data representative of the population of miners (N = 3375) in the surveyed camps across the study regions.

## Results

### Description of study population

Miners were predominantly male (87%); with significantly higher males in Region 7 (90%) compared with Region 1 (83%) and Region 8 (84%). The average age of miners was 36 years with a range of 18 to 82 years. There was no difference in the distribution of ages between regions. Christianity was the predominant religious belief reported by miners (80%) and this was significantly lowest in Region 1 (77%). Less than half of the miners (41%) were married or cohabitating with a partner and this was significantly higher in Region 7 (46%). About two-thirds (66%) of miners had a post primary education while about a quarter (28%) had a post-secondary education. Miners in Region 7 had higher levels of education compared to other regions.

The average number of miners per camp was 20 (range: 1–456, across small to large camps) and this was similar across regions. Overall, miners reported an average mining experience of nine years and this was highest in Region 7 (10 years) compared with other regions. On average, miners had worked for less than two years in the camp where they were surveyed, and this was uniform across regions. Overall, a third (33%) of miners worked in 12-month intervals and this was lowest in Region 8 (26%). Table 2 provides a detailed description of the study population.

**Table 2:** Description of study population.

Characteristics	Region 1	Region 7	Region 8	Total	P value
Total N (%)	390 (17.51)	819 (55.76)	476 (26.73)	1685 (100)	
Male* N (%)	321 (82.85)	716 (89.59)	397 (83.60)	1434(86.81)	0.018
Christian N (%)	301 (77.43)	690 (81.48)	372 (78.20)	1363 (79.90)	0.359
Average age in years Mean (SD)	34.98 (13.30)	36.33 (12.34)	35.87 (12.33)	35.97 (12.51)	0.268
% Married* N (%)	133 (34.81)	370 (45.51)	170 (35.71)	673 (41.02)	0.024
% with Post-primary education** N (%)	208 (52.58)	596 (71.82)	296 (62.26)	1100 (65.90)	<0.001
% with Post-secondary education*** N (%)	76 (20.01)	267 (30.67	125 (26.15)	468 (27.60)	0.004
Average # of miners at camp Mean (SD)	19.43 (11.15)	21.17 (35.28)	17.68 (10.85)	19.93 (27.36)	0.080
Average # of years as a miner. Mean (SD)	6.23 (8.08)	10.11 (9.67)	7.59 (8.52)	8.76 (9.24)	>0.001
Average # years spent at current camp. Mean (SD)	1.97 (4.03)	1.50 (2.89)	1.60 (3.70)	1.61 (3.34)	0.110
Proportion of miners who work all year** N (%)	133 (31.62)	312 (37.36)	123 (25.97)	568 (34.31)	0.003

Abbreviations: N = number SD = standard deviation.

* p≤0.05

**p ≤0.01

***p≤0.001 significant difference across regions.

All percentages, means and standard deviations represent weighted values.

It should be noted that not all of these demographic differences across regions are programmatically or epidemiologically important even though they are statistically significant. The sample size was determined based on the expectation that a subsequent study would evaluate the impact of a planned communication campaign to reduce miner’s self-treatment for malaria. We wanted to be able to detect a five-percentage point cross-sectional difference in this outcome variable at two points in time. This sample size decision increased the possibility of finding statistically significant differences for numerous variables. For the most important outcome variable that the communication campaign aims to address, the five-percentage point change target was based on average effect sizes that are typical of health communication campaigns. For example, in 2014 Snyder and LaCroix found that most campaigns produced changes in the range of 3–15 percentage points, with campaigns for shorter term or one time actions producing larger effect sizes than those for longer term or sustained behavior change [24]. Because medical malaria treatment and adherence to new drug regimens flies in the face of personal and cultural traditions, we opted for a relatively modest target of a five percentage point change.

### Key malaria-related behavior

Table 3 presents malaria testing and treatment behavior among miners. Forty percent of miners reported owning a mosquito net. Of these, 84% used a mosquito net the night preceding the survey. Thus, among all miners, a third (34%) used a mosquito net the night preceding the survey. Less than half (45%) of all miners surveyed had a fever in the past 12 months, and this did not differ significantly across regions. Across regions, two thirds (66%) of miners with a fever in the past 12 months sought care for their last fever episode. However, prompt care-seeking was much lower, with only 36% of miners seeking care the same day or next day of the onset of the fever episode. Over half (54%) of miners with fever in the past 12 months claimed that they self-medicated in their last episode and this was significantly lower in Region 1 and 8 (46% each) compared to Regions 7 (59%). About half (48%) of miners with fever in the past 12 months were tested during their fever episodes. This was lowest in Region 7 (43%), compared to Regions 1 (56%) and 8 (54%). Overall, 30% of miners with fever in the past 12 months tested positive for malaria during their last episode of fever, and this did not differ across regions. Among miners with fever in the past 12 months who tested positive for malaria during their last episode of fever, the majority (91%) were prescribed medications. Almost all (90%) of the miners who were prescribed medications claimed they took the prescribed medications. However, slightly less of these miners with fever (81%) completed their prescribed treatment regimen.

**Table 3:** Malaria testing and treatment among miners.

Among all Miners	Region 1 N (%)	Region 7 N (%)	Region 8 N (%)	Total N (%)	P value
Total	**390**	**819**	**476**	**1685**	
Miners that had a mosquito net*	140 (35.50)	303 (38.73)	216 (45.57)	659 (39.99)	0.021
Miners that slept under a mosquito net the previous night*	126 (32.17)	241 (31.54)	187 (39.50)	554 (33.78)	0.03
Miners that had a fever in the past 12 months**	157 (41.54)	386 (47.91)	202 (42.56)	745 (45.37)	0.007
**Among Miners with a Fever in the Past 12 Months**					
Total	**157**	**386**	**202**	**745**	
Miners that sought care for fever	108 (67.26)	248 (65.15)	134 (66.34)	490 (65.79)	0.906
Miners that sought care within the same day or next day of fever episode	49 (31.71)	146 (37.93)	72 (37.93)	267 (36.32)	0.424
Miners that self- medicated***	75 (46.03)	233 (59.29)	93 (45.95)	401 (53.82)	0.001
Miners that got tested for malaria*	89 (55.81)	168 (43.42)	110 (54.24)	367 (48.12)	0.023
Miners that tested positive for malaria	50 (31.68)	115 (29.66)	62 (30.65)	227 (30.23)	0.099
**Among Miners who Tested Positive for Malaria**					
Total	**50**	**115**	**62**	**227**	
Miners prescribed treatment after results	43 (84.21)	107 (94.62)	55 (88.79)	205 (91.39)	0.067
Miners that received prescribed medications	43 (84.21).	105 (93.37)	54 (87.10)	202 (90.24)	0.225
Miners that completed prescribed medications	39 (75.39)	86 (81.43)	52 (83.87)	177 (81.04)	0.126

Abbreviations: n = number

* p≤0.05

**p ≤0.01

***p≤0.001 significant difference across regions.

All percentage values have been weighted.

### Factors associated with malaria prevention- net use among miners with mosquito net

The results presented in Table 4 below show factors associated with mosquito net use after adjusting for potential demographic and psychosocial variables. Mosquito net use among miners with mosquito nets was significantly lower in Region 7 (adjusted odds ratio (aOR) 0.40; 95% confidence interval (CI): 0.17, 0.93), compared to Region 1. In contrast, mosquito net use among miners with mosquito nets was three times higher among miners who believed that most of their friends/coworkers with mosquito nets used them compared to those who did not (aOR: 3.11; 95% CI:1.65, 5.88). In addition, miners with high malaria knowledge had a higher mosquito net use (aOR: 1.92; 95% CI:1.19, 3.10). While no direct effect between SBC exposure and self-reported mosquito net use was seen, an indirect effect mediated by the association between SBC exposure and malaria knowledge was observed. Of note, no formal SBC campaign was implemented as of the time of the survey.

**Table 4:** Factors sssociated with mosquito net use among miners with a mosquito net (n = 659).

Factors	% that used a net	OR^a^ (95% CI)	aOR^a^ (95% CI)
Region			
Region 1	90.63	1.00 (n/a)	1.00 (n/a)
Region 7	81.42	**0.45 (0.21, 0.96)**	**0.40 (0.17, 0.93)**
Region 8	86.67	0.67 (0.32, 1.43)	0.71 (0.31, 1.62)
Sex			
Female (reference)	90.80*	1.00 (n/a)	1.00 (n/a)
Male	83.40	**0.51 (0.26, 1.01)**	0.66 (0.33, 1.34)
Religion			
Non-Christian (reference)	83.32	1.00 (n/a)	1.00 (n/a)
Christian	84.72	1.11 (0.54, 2.27)	1.10 (0.57, 2.11)
Age			
Less than 35 years (reference)	81.66	1.00 (n/a)	1.00 (n/a)
35 years or more	87.58	1.58 (0.96, 2.60)	1.57 (0.93, 2.66)
Marital Status			
Not Married (reference)	82.58	1.00 (n/a)	1.00 (n/a)
Married	87.00	1.41 (0.88, 2.27)	1.43 (0.85, 2.43)
Secondary education			
No (reference)	85.24	1.00 (n/a)	1.00 (n/a)
Yes	82.32	0.81 (0.48, 1.36)	0.74 (0.43, 1.27)
Length of time as a miner			
Less than five years (reference)	84.79	1.00 (n/a)	1.00 (n/a)
Five years or more	84.12	0.95 (0.56, 1.61)	0.84 (0.51, 1.38)
Perceives that most or all miners with mosquito nets use them			
No (reference)	75.22	1.00 (n/a)	1.00 (n/a)
Yes	89.80	**2.89 (1.64, 5.13)**	**3.11 (1.65, 5.88)**
High malaria knowledge score			
No (reference)	80.87***	1.00 (n/a)	1.00 (n/a)
Yes	90.00	**2.13 (1.36, 3.34)**	**1.92 (1.19, 3.10)**
High malaria belief score			
No (reference)	82.75	1.00 (n/a)	1.00 (n/a)
Yes	86.87	1.38 (0.81, 2.35)	1.15 (0.70, 1.90)

Abbreviations: aOR = adjusted odds ratio, n = number, OR = odds ratio

* p≤0.05

**p ≤0.01

***p≤0.001 significant difference across characteristics.

Covariates in the model included region, sex, religion, age, marital status, education level, mining experience and exposure to malaria prevention messages, number of confirmed malaria episodes, malaria knowledge score, malaria belief score and norms related to net use.

### Factors associated with malaria care seeking, testing and treatment

Table 5 presents the factors associated with care-seeking among miners with a fever in the past 12 months. Prompt care-seeking was higher among miners with a high level of malaria knowledge (aOR: 1.44; 95% CI: 1.01, 2.05).

**Table 5:** Factors associated with care-seeking among miners with a fever in the past 12 months.

Behavior	Prompt care-seeking		Malaria Testing		Self-Medication	
Characteristics	(%)	aOR^a^ (95% CI)	(%)	aOR^a^ (95% CI)	(%)	aOR^a^ (95% CI)
**Total**	**35.84**		**49.26**		**53.83**	
Region						
Region 1(reference)	31.71	1.00 (n/a)	55.81*	1.00 (n/a)	46.03***	1.00 (n/a)
Region 7	37.93	1.33 (0.84, 2.09)	43.42	**0.54 (0.36, 0.80)**	59.29	**1.76 (1.21, 2.58)**
Region 8	35.50	1.22 (0.80, 1.84)	54.24	1.05 (0.67, 1.66)	45.95	1.06 (0.75, 1.51)
Sex						
Female (reference)	40.04	1.00 (n/a)	57.91*	1.00 (n/a)	67.90**	1.00 (n/a)
Male	35.71	0.84 (0.52, 1.37)	46.50	0.74 (0.47, 1.15)	51.49	**0.52 (0.32, 0.86)**
Religion						
Non-Christian (reference)	38.62	1.00 (n/a)	44.94	1.00 (n/a)	47.43	1.00 (n/a)
Christian	35.81	0.82 (0.51, 1.31)	48.83	1.04 (0.65, 1.68)	55.25	1.26 (0.72, 2.21)
Age						
Less than 35 years (reference)	38.07	1.00 (n/a)	47.10	1.00 (n/a)	49.55	1.00 (n/a)
35 years or more	34.05	0.82 (0.53, 1.25)	49.44	1.11 (0.71, 1.74)	59.39	1.33 (0.88, 2.01)
Marital Status						
Not Married (reference)	35.62	1.00 (n/a)	45.86	1.00 (n/a)	49.85*	1.00 (n/a)
Married	37.29	1.07 (0.71, 1.62)	51.24	1.26 (0.90, 1.77)	59.31	1.21(0.85, 1.72)
Secondary education						
No (reference)	33.42	1.00 (n/a)	43.50***	1.00 (n/a)	52.57	1.00 (n/a)
Yes	42.77	1.34 (0.83,2.15)	58.36	**1.71 (1.16, 2.51)**	56.59	1.08 (0.77, 1.50)
Length of time as a miner						
Less than five years (reference)	38.41	1.00 (n/a)	49.39	1.00 (n/a)	52.36	1.00 (n/a)
Five years or more	34.21	0.87 (0.62, 1.23)	46.83	0.93 (0.68, 1.29)	55.30	1.05 (0.73, 1.51)
Prior episode of confirmed malaria						
No (reference)	31.82	1.00 (n/a)	32.37***	1.00 (n/a)	52.24	1.00 (n/a)
Yes	38.59	1.29 (0.74, 2.26)	56.05	**2.61 (1.71, 4.00)**	54.62	1.04 (0.76, 1.44)
High malaria knowledge score						
No (reference)	32.52*	1.00 (n/a)	43.49**	1.00 (n/a)	50.18	1.00 (n/a)
Yes	41.83	**1.44 (1.01, 2.05)**	54.83	**1.45 (1.02, 2.05)**	59.09	1.33 (0.87, 2.02)
High malaria belief score						
No (reference)	35.71	1.00 (n/a)	44.39*	1.00 (n/a)	56.53	1.00 (n/a)
Yes	37.38	1.08 (0.75, 1.54)	54.58	**1.59 (1.12, 2.27)**	49.13	0.70 (0.45, 1.08)

Abbreviations: aOR = adjusted odds ratio, n = number, n/a = not applicable OR = odds ratio

* p≤0.05

**p ≤0.01

***p≤0.001 significant difference across regions.

Adjusted for region, sex, religion, age, marital status, education level, mining experience and exposure to malaria prevention messages, number of confirmed malaria episodes, malaria knowledge score and malaria belief score.

Malaria testing rates were significantly lower in Region 7, compared to Region 1 (aOR: 0.54; 95% CI: 0.36, 0.80). In addition, testing rates were significantly higher among miners with secondary education (aOR: 1.71; 95% CI: 1.16, 2.51). In addition, testing rates were significantly higher among miners with prior episodes of confirmed malaria (aOR: 2.61; 95% CI:1.71, 4.00) and those with high malaria knowledge (aOR: 1.45; 95% CI: 1.02, 2.05) and malaria belief (aOR:1.59; 95% CI: 1.12, 2.27).

Self-medication was significantly higher among miners in Region 7 compared to Region 1 (aOR: 1.76; 95% CI: 1.21, 2.58). However, male miners were significantly less likely to self-medicate than female miners (aOR: 0.52; 95% CI: 0.32, 0.86).

## Discussion

This research explored the prevalence and factors influencing self-reported malaria care-seeking and treatment behaviors in the gold mining camps of Regions 1, 7, and 8 in Guyana in the context of a recent mosquito net distribution campaign in 2018 and the pilot and scale-up of a community case management initiative since 2016. Mosquito net use was low overall but high among miners who owned a mosquito net. In addition, mosquito net use among miners who owned mosquito nets was higher among miners who believed that net use was the norm in their camp. The World Health Organization (WHO) recommends the consistent use of insecticide treated mosquito nets as a major strategy to prevent malaria. While no direct effect between SBC exposure and self-reported mosquito net use was seen, an indirect effect mediated by the association between SBC exposure and malaria knowledge was observed. These findings are consistent with other literature demonstrating the influence of malaria knowledge and SBC messaging on mosquito net use [25–28]. Of note, no formal SBC campaign had been implemented prior to data collection and respondents probably recalled ad-hoc messages by the MoH. Access to a mosquito net has been demonstrated as the most important factor influencing net use [26,29]. In this population, most miners have lived in the mining area for more than a year and should have received mosquito nets during the last national distribution campaign in 2018. Additional efforts such as a post ITN distribution assessment may be needed to verify that mining camps were reached during the last mosquito net distribution campaign and address potential bottlenecks, thereby improving coverage to mosquito nets among this high-risk population. Further qualitative research should aim to understand and address the reasons why miners who do have nets do not use them. Mass distributions of ITNs tend to occur periodically (every 3–5 years) and are typically complemented by continuous distribution channels within the public or private sector [30]. In Guyana, the health sector is a continuous distribution channel for ITNs as they can be obtained free of charge from a health center by miners or their camp managers. Additional efforts may be needed to ensure that this policy is widely disseminated and that miners are aware of the nearest ITN distribution channels. Tailored SBC messaging on net acquisition, use and care will be beneficial to promoting a culture of consistent net use among miners.

In this study, the prevalence of self-reported history of fever among all miners as well as the proportion of miners who reported that they subsequently tested positive for malaria did not vary significantly across the regions, which suggests that malaria risk may be comparable across the study regions [31]. Self-reported prompt care-seeking and testing for malaria was generally low but highest among miners with prior malaria episodes and those with a more comprehensive knowledge regarding malaria. On the other hand, reported self-medication rates were somewhat high among miners. The lowest rates of reported self-medication were seen among miners with a more comprehensive knowledge regarding malaria. The lower self-reported malaria testing and self-medication rates observed in male miners compared to female miners warrants further investigation. Observed regional differences such as the lower self-reported testing and higher reported self-medication rates in Region 7 suggest that there may be contextual factors influencing these outcomes which may need to be explored further. For example, Region 8 anecdotally has a more accessible road network and can be traversed easily compared to the other regions. Although the community case management initiative was not piloted in Region 1, significantly more miners from Region 1 reported that they sought testing for malaria. This may be a result of government health centers in close proximity to the main mining camps, thus providing better access to health care, than Regions 7 and 8 [3,31–33].

In addition to strategies to prevent malaria, prompt and effective case management has been promoted by the WHO as a key malaria intervention to prevent morbidity, mortality and continuous malaria transmission. Relevant behavior for effective malaria case management among miners includes miners seeking appropriate care within 24 hours of experiencing symptoms without prior self-medication, getting tested for malaria, and if positive, adhering to the treatment regimen. Study findings suggest additional efforts are needed to improve prompt care-seeking and testing rates. While many miners sought appropriate care, few of them sought appropriate care promptly per the national treatment guidelines. Miners may lack appropriate awareness on the need for prompt care-seeking or malaria testing as they perceive their symptoms to not be severe. Miners may also face other barriers to prompt care seeking and malaria testing, such as the cost of transportation, lack of time to seek testing services and other costs in terms of lost wages when taking time off work to get tested [33].

With Guyana’s roll out of the community case management initiative, malaria testing and treatment locations should be more accessible to miners in the future. Appropriate signage and billboards may be used to advertise these test locations and may be complemented with supporting print media such as treatment adherence handouts for the miners and counselling guides for the testers. The initiative may also benefit from additional SBC efforts such as community mobilization and engagement, mass media campaigns to sensitize miners about the testing locations and the importance of prompt care seeking and other relevant behavior. However, this needs to be complemented by additional efforts such as supply chain management of commodities, continuous training and support to ensure that the volunteer testers have the necessary technical and communication skills. Incentives in cash or kind to the volunteer testers may also promote retention rates among the testers while public private partnerships between the MoH and relevant mining organizations may ensure the overall sustainability of the community case management initiative.

The high rate of self-medication among miners has been noted in mining communities of Africa and Latin America, perhaps due to miners’ presumptions about their symptoms and their perceived knowledge of “what works” to mitigate such symptoms such as alternative or over-the-counter medications [34]. Nonetheless, the high self-reported rates of self-medication remain concerning since self-medication can be considered a proxy for not seeking appropriate care [33]. Such medications are usually traditional or herbal concoctions or formal medications obtained from pharmacies or shops that lack licensed or accredited health service providers [12]. Furthermore, such medications are given without proper diagnostic tests being carried out [18]. Finally, self-medication may also influence the validity of malaria test results and immunological processes [35]. A recent qualitative study demonstrated that miners who chose to initiate self-treatment—using unregulated medications from the private and informal sector—did so out of convenience and the belief that self-treatment had worked before. Miners who completed the full government-approved treatment understood the need to complete the treatment, while those who prematurely stopped treatment did so because of medication side effects and a desire to feel better as soon as possible [36]. There is a great need for communication interventions to stress the dangers of self-medication alongside the need for prompt and appropriate care-seeking. Thus, SBC messaging may be beneficial in addressing gaps in knowledge, attitudes, and perceptions and might play an essential role in ensuring the adoption of relevant behavior such as prompt care-seeking, malaria testing, and adherence to medications [37,38].

This study has some limitations. The sample size calculations did not include a design effect to account for clustering at the camp level. Also, the data is based on miners’ self-report during face to face interviews which may be prone to recall and social desirability bias. In addition, the cross-sectional study design does not permit inferences of causality. Our sampling methodology only samples miners who were present on the day of data collection. We do not have any information regarding the miners who were not in camp and thus did not have the opportunity to be recruited into the study. However, while this is yet to be validated, we do not envisage major differences in the demographic and/or psychosocial characteristics of the miners sampled versus those not sampled and thus do not assume any major biases due to this. In addition, the survey was not attempted in a few areas due to prohibitive transportation costs and it is unclear to what extent such areas might differ from the final study areas. It is possible that the hard to reach camps not included in the study may be less likely to seek care for malaria and have difficulty maintaining supply chains needed to run the community case management program, potentially resulting in reduced malaria care-seeking and testing rates. Finally, while we adjusted for contextual issues or known confounders, unmeasured or unknown confounding may still be an issue. Our findings may also be influenced by structural issues. Stock out of RDTs, for example, might mask differential malaria testing rates observed.

## Conclusion

This cross-sectional study aimed to explore factors influencing malaria prevention and care-seeking behaviors among gold miners in Guyana. Mosquito net use, prompt care-seeking and malaria testing were low while self-medication was high across all the study regions. Factors influencing these behaviors included ownership of a mosquito net, perceived norms regarding mosquito net use, comprehensive knowledge about malaria, experience of prior episode of confirmed malaria, and positive beliefs/attitudes towards malaria. Study findings have implications for malaria interventions in Guyana such as the mass and continuous distribution of ITNs as well as the ongoing community case management initiative using trained malaria testing and treatment volunteers to curb malaria transmission among remote gold mining populations.

## Supporting information

S1 Table Estimated vs. verified numbers of miners in Regions 1, 7, and 8, as of November 17, 2019. (DOCX)

S2 Table Sampling implementation summary showing number of mining camps and miners verified, approached and interviewed. (DOCX)

S1 Dataset (DTA)
