# Supplementary material for: Malaria prevention and care seeking among gold miners in Guyana
Source: PLoS One. 2020 Dec 29;15(12):e0244454. doi: 10.1371/journal.pone.0244454 (PMC7771697; doi:10.1371/journal.pone.0244454)
Supplement: S1 Table — (DOCX) [file pone.0244454.s001.docx]

# Malaria prevention and care seeking among gold miners in Guyana

# Supporting Information

**S2 Table. Sampling implementation summary showing number of mining camps and miners verified, approached and interviewed.**

| **District** | **# estimated miners from verification exercise*** | **# of camps surveyed** | **# active miners in surveyed camps** | **# miners approached** | **# of miners interviewed** |
| --- | --- | --- | --- | --- | --- |
| **REGION 1** |  |  |  |  |  |
| MATTHEW’S RIDGE | 298 | 16 | 218 | 185 | 185 |
| ARAKAKA | 121 | 12 | 139 | 99 | 97 |
| BARAMITA | 298 | 6 | 151 | 64 | 64 |
| PORT KAITUMA | 78 | 8 | 83 | 45 | 44 |
| **Total** | **795** | **42** | **592** | **393** | **390** |
| **REGION 7** |  |  |  |  |  |
| LOWER MAZARUNI | 699 | 10 | 584 | 67 | 67 |
| MIDDLE MAZARUNI* | 368 | 33 | 382 | 272 | 272 |
| POTARO ROAD | 562 | 4 | 61 | 37 | 37 |
| CUYUNI RIVER** | 857 | 71 | 855 | 443 | 443 |
| **Total** | **2486** | **118** | **1882** | **819** | **819** |
| **REGION 8** |  |  |  |  |  |
| SUB-REGION 1 | 296 | 34 | 445 | 247 | 246 |
| SUB-REGION 2* | 474 | 39 | 457 | 231 | 230 |
| **Total** | **770** | **73** | **902** | **478** | **476** |
|  |  |  |  |  |  |
| **GRAND TOTAL** | **4051** | **233** | **3375** | **1690** | **1685** |
| The verification exercise could not be conducted in 28 areas In Region 1, eight areas (Yakarika, Barima Kariabo, Kokerite Backdam, Yakashuri Backdam, Pipianni Backdam, Ianna Backdam, Aranka and White Creek) were not visited due to due to prohibitive transportation costs. In Region 7, a total of 14 areas were not verified. Five areas (Turtle Creek, Olive Creek, Looking glass Falls, Kurupung - K3, Kurupung – Crappo Rock) were inaccessible; four areas (Arau, Mango Landing, San Juan and Eteringbang Falls) were not visited due to security concerns while five areas in Upper Mazaruni (Imbamaidai Backdam, Jawalla Backdam, Chi – Chi Falls, Ekereku and Kamarang) were not visited due to prohibitive transportation costs. In Region 8, one area (Maikwak) in Sub-Region 1 and five areas in sub-Region 2 (Echerak, Chenapau, Monkey Mountain, Kato and Iuwang) were not visited due to prohibitive transportation costs | | | | | |
